# Silencing of Fused Toes Homolog (FTS) Increases Radiosensitivity to Carbon-Ion Through Downregulation of Notch Signaling in Cervical Cancer Cells

**DOI:** 10.3389/fonc.2021.730607

**Published:** 2021-10-26

**Authors:** Prabakaran D.S., Pankaj Kumar Chaturvedi, Takashi Shimokawa, Ki-Hwan Kim, Woo-Yoon Park

**Affiliations:** ^1^Department of Radiation Oncology, Chungbuk National University Hospital, Chungbuk National University College of Medicine, Cheongju, South Korea; ^2^Department of Accelerator and Medical Physics, Institute for Quantum Medical Science, QST, Chiba, Japan; ^3^Department of Radiation Oncology, Chungnam National University Hospital, Daejeon, South Korea

**Keywords:** cervical cancer, fused toes homolog, notch, spheroid, carbon-ion beam

## Abstract

The effects of Carbon ion radiation (C-ion) alone or in combination with fused toes homolog (FTS) silencing on Notch signaling were investigated in uterine cervical cancer cell lines (ME180 and CaSki). In both cell lines, upon irradiation with C-ion, the expression of Notch signaling molecules (Notch1, 2, 3 and cleaved Notch1), γ-secretase complex molecules and FTS was upregulated dose-dependently (1, 2 and 4 Gy) except Notch1 in ME180 cells where the change in expression was not significant. However, overexpression of these molecules was attenuated upon silencing of FTS. The spheroid formation, expression of stem cell markers (OCT4A, Sox2 and Nanog) and clonogenic cell survival were reduced by the combination as compared to FTS silencing or C-ion irradiation alone. Additionally, immunoprecipitation and immunofluorescence assay revealed interaction and co-localization of FTS with Notch signaling molecules. In conclusion, FTS silencing enhances the radio-sensitivity of the cervical cancer cells to C-ion by downregulating Notch signaling molecules and decreasing the survival of cancer stem cells.

## Introduction

Uterine cervical cancer is the fourth most commonly diagnosed cancer with the fourth leading cause of death due to cancer in women worldwide. There were estimates of 604,000 new cases and 342,000 deaths in 2020. Nevertheless, it is the leading cause of malignancies related death in sub-Saharan Africa, South America and South-Eastern Asia (1). The human papillomavirus (HPV), most frequently HPV 16, is the predominant risk factor of cervical cancer (2). Notch signaling has been reported to play a crucial role in cervical cancer development (3) in which E6 and E7 oncoproteins of HPV regulate the Notch expression and conjoin to induce cellular transformation (4).

Notch signaling is a conserved pathway that determines mammalian cell fate (5). The transmembrane receptors of Notch communicate with the neighboring cells which express membrane-bound ligands. The interaction of Notch ligands with its receptors triggers proteolytic cleavage leading to the release and translocation of the Notch intracellular domain (NICD) to the nucleus where NICD activates the target genes transcription. Constitutive Notch signaling targets comprise not only the transcriptional regulators of the hairy enhancer split (HES) and HES related with YRPW motif-family (HEY), but also the oncogenes like Myc or Ras (6). Thus, the Notch pathway plays a central role in the maintenance of cancer stem-like properties and its persistent activation may lead to cancer progression and metastasis (7, 8).

Even though some tumors relapse, radiation therapy (RT) is considered as one of the main modalities for cancer treatment (9). Recently, Notch pathway has been demonstrated to mediate resistance for RT in tumor cells (10). A thorough understanding of Notch regulation and its interactions with other relevant therapeutic pathways is essential for its successful targeting (11).

Carbon ion (C-ion) RT offers more advantages than conventional RT as it enables efficient cell killing, attributable to a more accurate dose distribution (12). Furthermore, C-ion allows high energy deposition and high linear energy transfer (LET) to its target compared with photon and proton beams. In addition, the upsurge of energy deposition along the path of ion beam in the body results in less toxicity to the neighboring normal tissues (13). More than 27,000 patients with various types of tumors, including adenoid cystic carcinoma, adenocarcinoma, malignant melanoma and sarcomas, which are very often x-rays resistant, have been treated with C-ion worldwide during 1994-2018 (14, 15).

A favorable local control with minimal radiation toxicity by C-ion RT alone has been reported for locally advanced cervical cancer (16). However, despite favorable local control rates, distant metastasis was high, disease-free survival and overall survival rates were not too satisfactory compared to concurrent chemoradiotherapy (CCRT) (17), therefore, efforts to improve the efficacy of C-ion therapy are highly desirable.

Cancer stem cells (CSCs) can self-renew, differentiate and repair DNA damage, which renders them resistant to various therapies, including RT (18). The dose-response curves comparison for the CSCs and non-CSCs indicated that CSCs are resistant to x-rays and C-ion beam (19). Therefore, eradication of all CSCs is a prerequisite for ultimate cancer treatment. Some researchers have reported that cisplatin or gemcitabine in combination with C-ion RT in pancreatic, mesothelioma and breast cancer improved the efficacy of C-ion and overcame CSC resistance (20–22). The invasive and migratory potential of CSCs in head and neck squamous cell carcinoma (HNSCC) enhance their radioresistance. CSC invasion process was significantly inhibited by the combination of cetuximab and C-ion (23). In uterine cervical cancer C-ion therapy overcame the radiation resistance origination from hypoxia (24). The radioresistance in CSCs has been often linked with Notch signaling. Thus inhibition of Notch pathway could be used to develop an adjuvant approach to RT (25).

Our previous findings have shown FTS as a potential target for Notch-mediated resistance upon x-ray irradiation in cervical cancer (10). In this study, effects of the C-ion beam on the Notch signaling and spheroid formation were investigated in FTS intact and silenced cells with an objective to evaluate whether targeting FTS can be a Notch-mediated adjuvant approach in improving the efficacy of C-ion therapy in cervical cancer.

## Materials and Methods

### Cell Culture and Antibiotics

Two human cervical cancer cell lines, ME180 and CaSki cells, were procured from RIKEN BioResource Center (Japan) and cultured in RPMI 1640 and DMEM (Invitrogen, Carlsbad, CA, USA) supplemented with 10% fetal bovine serum (FBS), penicillin (100 units/mL) and streptomycin (100 mg/mL). The cells were grown in a humidified incubator at 37°C and 5% CO_2_. Cell harvesting and passaging was done with the help of Trypsin-EDTA. All standard cell culture reagents were procured from Invitrogen. Antibodies for FTS and Actin were purchased from Santa Cruz Biotechnology, Inc. (Dallas, TX, USA). All other primary and secondary antibodies were purchased from Cell Signaling Technologies (Beverly, MA, USA).

### C-Ion and X-Ray Beam Irradiation

Cells grown in T-25 flasks were irradiated at room temperature with C-ions accelerated by the Heavy Ion Medical Accelerator in Chiba (HIMAC) at the National Institute of Radiological Sciences (NIRS) (Chiba, Japan). The 290 MeV/n carbon-ion beams were adjusted to be about 70 KeV/µm at the cell surface using a scatterer (Ta = 0.2 mm, Pb = 1.6 mm), air: 11.8 m, the flask and PMMA range shifters: 140 mm water-equivalent. The flasks were positioned in vertical position with the cell adhesion surface facing the beam source. The particulars regarding the beam characteristics of C-ion, dosimetry and irradiation procedures have been explained previously (26–28). To compare the radiation effects of C-ion and x-ray by FTS silencing, cells were also irradiated with various doses of x-ray in a field size of 20 cm x 20 cm at room temperature using a 6 MV medical linear accelerator (Oncor, Siemens, Concord, CA, USA) at Chungbuk National University Hospital, Department of Radiation Oncology. QA of the medical linear accelerator is performed in compliance to IAEA-TRS-398.

### Colony Formation Assay

The cell survival by irradiation was evaluated using colony formation assay. After C-ion/x-ray irradiation, cells were washed with 1X PBS pH 7.5 and single-cell suspension was prepared by trypsinization. The cells were counted and re-seeded in triplicates into 60 mm cell culture dishes at appropriate cell densities for colony formation. The cells were cultured for 7-9 days to make colonies, fixed with 20% ethanol and stained with 0.2% crystal violet (Sigma, St. Louis, Missouri, USA). The colonies comprising 50 or more cells were considered as survivors and counted using a microscope (Olympus Optical, Shinjuku, Tokyo, Japan). Surviving fractions were calculated on the basis of the plating efficiencies of corresponding non-irradiated cells. Three independent experiments were performed with each cervical cancer cell line. The graphs were plotted for surviving fractions by C-ion and x-ray in the FTS intact and silenced cells. Dose modifying factor (DMF) was calculated as the ratio between FTS intact and silenced group for the radiation doses of C-ion or x-ray with 10% surviving fraction (SF_10_).

### Western Blotting

After irradiation, the cells were washed in cold PBS and lysed with 200 µl of cell lysis buffer (Cell Signaling Technology, Danvers, MA, USA) supplemented with complete protease inhibitor cocktail and phosphatase inhibitors (Roche, Mannheim, Germany) for 30 min on ice. Protein concentration of each sample was determined using Bradford reagent (Bio-Rad, Hercules, CA, USA). 30 µg of total protein from each sample was resolved on SDS-PAGE gels and transferred onto PVDF membranes (Millipore, Billerica, MA, USA). The membranes were then probed with appropriate primary and secondary antibodies. Finally, the membranes were exposed to the ECL substrate solution (Thermo Scientific, Rockford, IL, USA) and images were recorded with the help of chemidoc (Fujifilm, Tokyo, Japan). Expression of each protein was calculated by densitometric measurement using the Multi-Gauge ver. 3.1 Software (Fujifilm, Tokyo, Japan). Band densities of target proteins were normalized to actin expression to plot the bar graphs (**Supplementary Figures S2–S5**).

### Spheroid Formation Assay

After transfection with scrambled siRNA or FTS siRNA for 24 h, the cells were irradiated with C-ion beam. Post-irradiation, the cells were trypsinized, harvested and single-cell suspension was prepared. The single-cell suspension was cultured in 60 mm ultralow attachment plates (Corning, Lowell, MA, USA), in a serum-free DMEM/F12 growth medium supplemented with 10 ng/mL EGF (Sigma, St. Louis, MO, USA), 10 ng/mL bFGF (Invitrogen) and 2% B27 (Invitrogen). The images of spheroids were taken using the Olympus IX71 microscope (Tokyo, Japan).

### Cell Viability Assay in Spheroids

To analyze the number of viable cells constituting the spheroids, 7 days after spheroid culture 50 µl of WST-1 solution (DoGenBio, Seoul, South Korea) was added to each well. After 4 h, absorbance at 450 nm was recorded with the help of a microplate reader (BIO-RAD, CA, USA).

### FTS Silencing

Cells were seeded in T-25 or T-75 flask in antibiotic-free RPMI and allowed to attach overnight. Next morning, the medium was replaced with transfection medium containing 50 nM of either scrambled siRNA (sc-37007, Santa Cruz) or FTS siRNA (sc-93013, Santa Cruz) and incubated at 37˚C. After 6 h, the medium was replaced with complete growth medium supplemented with 10% FBS and 1% antibiotics, and the cells were incubated for another 24 h.

### Immunoprecipitation (IP) Assay

The cells were lysed 24 h after C-ion irradiation using cell lysis buffer (Cell Signaling Technology) for 30 min on ice and scraped using cell scraper (SPL Life Sciences, South Korea). The cell lysates were centrifuged at 12,000 g for 10 min at 4°C and the supernatants were collected. 200 μg of total protein from each sample was incubated overnight with the anti-FTS antibody at 4°C, followed by incubation with protein A/G agarose (Santa Cruz) for 1 h. Immunoprecipitates were washed twice for 5 min with cell lysis buffer at 4°C. Bead bound proteins were eluted with non-reducing sample buffer (Thermo Scientific) at 95°C for 3 min and then subjected to SDS-PAGE and western blot analyses.

### Immunofluorescence (IF) Assay

Cells were grown on chamber slides (154526, Thermo Fisher, MA, USA), transfected using scrambled or FTS siRNA, irradiated with 1 Gy C-ion and incubated for 24 h at 37°C in a CO_2_ incubator. After incubation, the cells were fixed with 4% formaldehyde (Thermo Scientific), permeabilized with 0.1% Triton X-100 (Amresco, Ohio, US) and blocked with 10% FBS for 30 min followed by overnight incubation at 4°C with respective primary antibody (1:100 dilution). Cells were further incubated in the dark at room temperature with Alexa-488/Alexa 594-conjugated secondary antibody for 1 h. Nuclei were counter stained with DAPI at a concentration of 1 μg/mL (Sigma). After staining with DAPI the chambers were removed and mounted using cover slips with anti-fade mounting solution (Dako, CA, US). The slides were dried overnight in the dark and stored at -20°C until imaging. Z-stack images were captured with the help of confocal microscope (Leica DM-IRB, Mannheim, Germany). For spheroids immunofluorescence, the cells were irradiated with 1 Gy C-ion after transfection with scrambled or FTS siRNA. The cells were cultured further in an ultra-low attachment plate and allowed to form spheroids. The spheroids were then carefully transferred onto chamber slides and allowed to adhere overnight. Next morning immunofluorescence protocol was followed as mentioned above for adherent cells.

### Statistical Analysis

All analytical data are presented as the means ± SD of three independent experiments. Differences among the groups were calculated by GraphPad Prism (GraphPad Software, version 9.1.0 (221), La Jolla, CA, USA, www.graphpad.com) using two way analysis of variance (ANOVA) module followed by Dunnett’s/Tukey multiple comparison post-hoc test; p ≤ 0.05, was considered to be statistically significant.

## Results

### C-Ion Upregulates FTS, the Notch Signaling and γ-Secretase Complex Molecules

**Figure 1:**
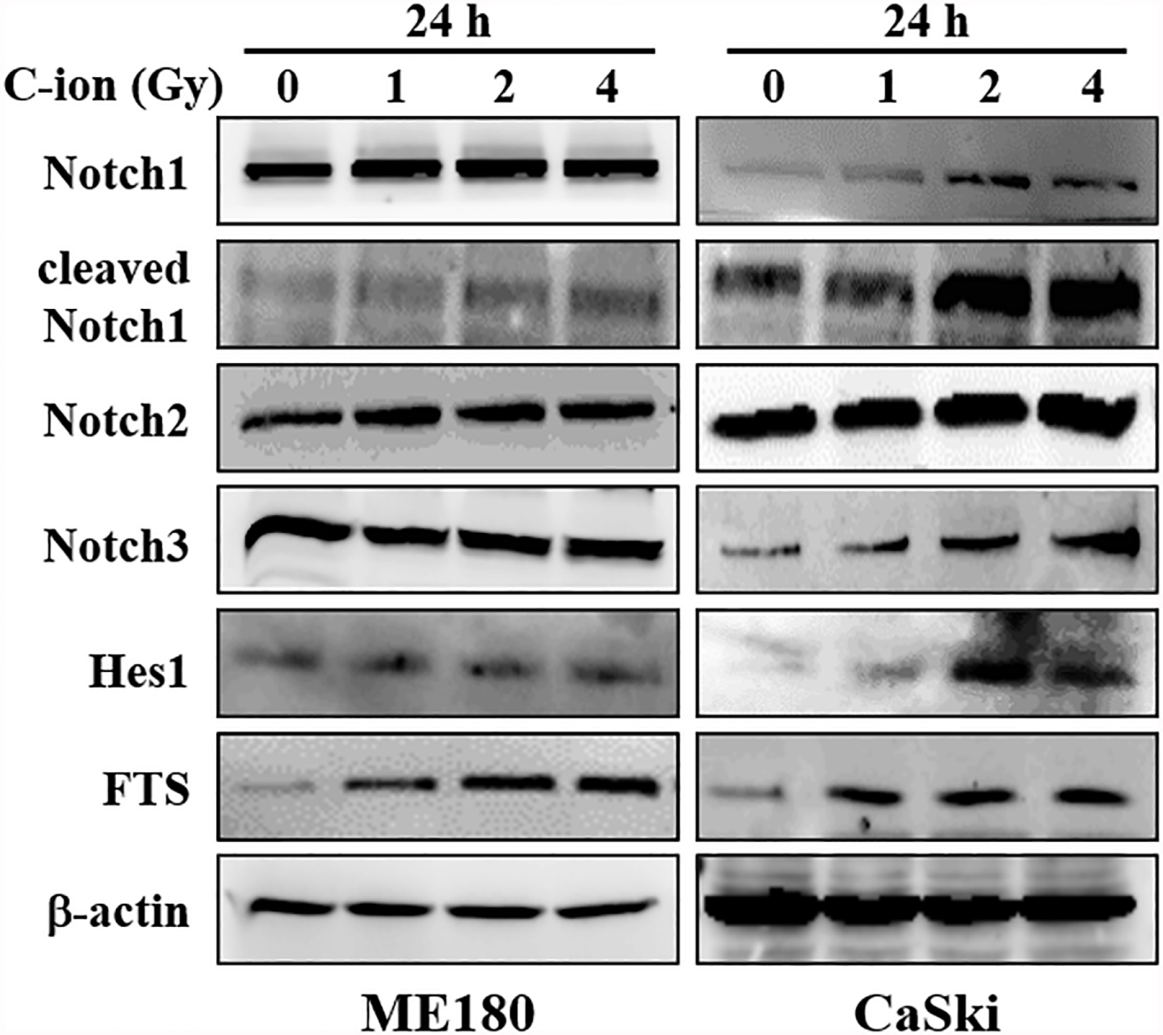
The expression of Notch signaling molecules (Notch1, 2, 3, cleaved Notch1, Hes1) and FTS were increased by C-ion beam in cervical cancer cells (ME180, CaSki). The cells were irradiated with 0, 1, 2 and 4 Gy of C-ion and the protein expression was measured with immunoblot 24 h post-irradiation. The images shown in this figure are the representatives of at least three independent experiments.

**Figure 2:**
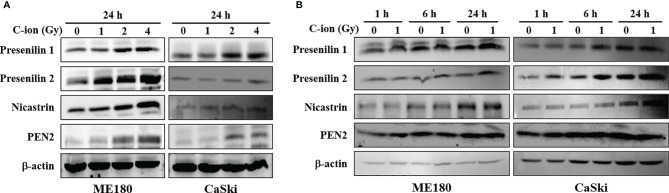
The expression of γ-secretase complex proteins was upregulated by C-ion. The cells were irradiated with 0, 1, 2 and 4 Gy of C-ion and the protein expression was detected by western blotting 24 h post-irradiation **(A)** or the cells were irradiated with 0 and 1 Gy C-ion and the protein expression was measured after 1, 6 and 24 h post-irradiation **(B)**. The images shown are the representatives of at least three independent experiments.

To determine the effect of C-ion on the Notch signaling, first, we investigated the change in the expression level of the Notch signaling molecules at three different doses of C-ion (0, 1, 2 and 4 Gy). In CaSki, the expression of Notch1, 2, 3, cleaved Notch1, Hes1 and FTS was increased dose-dependently in response to C-ion irradiation. In ME180, cleaved Notch1 and FTS expression increased dose-dependently, while other molecules expression didn’t change much in response to increased radiation doses (**Figure 1**). Similarly, the protein expression level of γ-secretase complex molecules (presenilin1, presenilin2, nicastrin and PEN2) was also elevated, in a dose and time-dependent manner in both cell lines (**Figures 2A, B**). Changes in the expression level of FTS, Notch and γ-secretase complex molecules in response to radiation doses were highly significant when compared with unirradiated group (**Supplementary Figures S2, S3**).

### FTS-Silencing Attenuates the Expression of Notch Signaling Molecules

**Figure 3:**
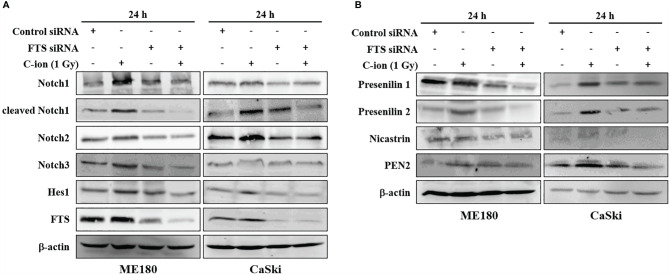
FTS-silencing combined C-ion radiation targets the protein expression of Notch molecules **(A)** and γ-secretase complex **(B)**. ME180 and CaSki cells were transfected with scrambled siRNA or FTS siRNA. Cells were irradiated with C-ion 1 Gy and the protein expression was measured with immunoblot 24 h post-irradiation. The images shown in this figure are the representatives of at least three independent experiments.

To study the role of FTS on Notch signaling, the FTS gene was silenced using siRNA. Silencing of FTS reduced C-ion induced upregulation of Notch1, 2, 3, cleaved Notch1 and Hes1 significantly (**Figure 3A** and **Supplementary Figure S4**), in addition to γ-secretase complex proteins (presenilin1, presenilin2, nicastrin and PEN2) (**Figure 3B** and **Supplementary Figure S4**) in both cell lines. Downregulation of these molecules was further augmented when FTS silencing was combined with C-ion irradiation (**Figures 3A, B**, and **Supplementary Figure S4**).

### Interaction Between FTS and the Notch Molecules Increases Upon C-Ion

**Figure 4:**
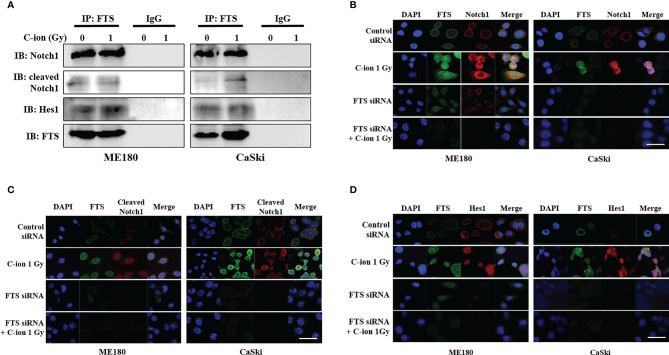
Immunoprecipitation assay and immunofluorescence show the interaction between FTS and Notch1/cleaved Notch1/Hes1 in ME180 and CaSki cells. Cells were irradiated with 0 or 1 Gy C-ion and lysed after 24 h. Immunoprecipitation was performed with whole cell lysates using FTS antibody. IgG was used as negative control **(A)**. For immunofluorescence, the cells were grown in chamber slides. Cells were irradiated with 0 or 1 Gy C-ion. FTS was detected with Alexa Fluor 488 (green), whereas Notch1, cleaved Notch1 and Hes1 were detected using Alexa Fluor 594 (red), 24 h post-irradiation **(B–D)**. The images shown in this figure are the representatives of at least three independent experiments. White bar in each panel corresponds to 10 µm.

Immunoprecipitation and immunofluorescence assay were performed to demonstrate the interaction between FTS and the Notch signaling molecules. Immunoprecipitation demonstrated the physical interaction between FTS and Notch1/cleaved Notch1/Hes1 (**Figure 4A**). Immunofluorescence showed the increased co-localization of FTS with Notch1/cleaved Notch1/Hes1 by C-ion irradiation, but the interaction and co-localization were reduced substantially by FTS-silencing (**Figures 4B–D**).

### The Combination of FTS-Silencing and C-Ion Decreases Spheroid Formation, Cancer Stem Cell Markers and Clonogenic Cell Survival

**Figure 5:**
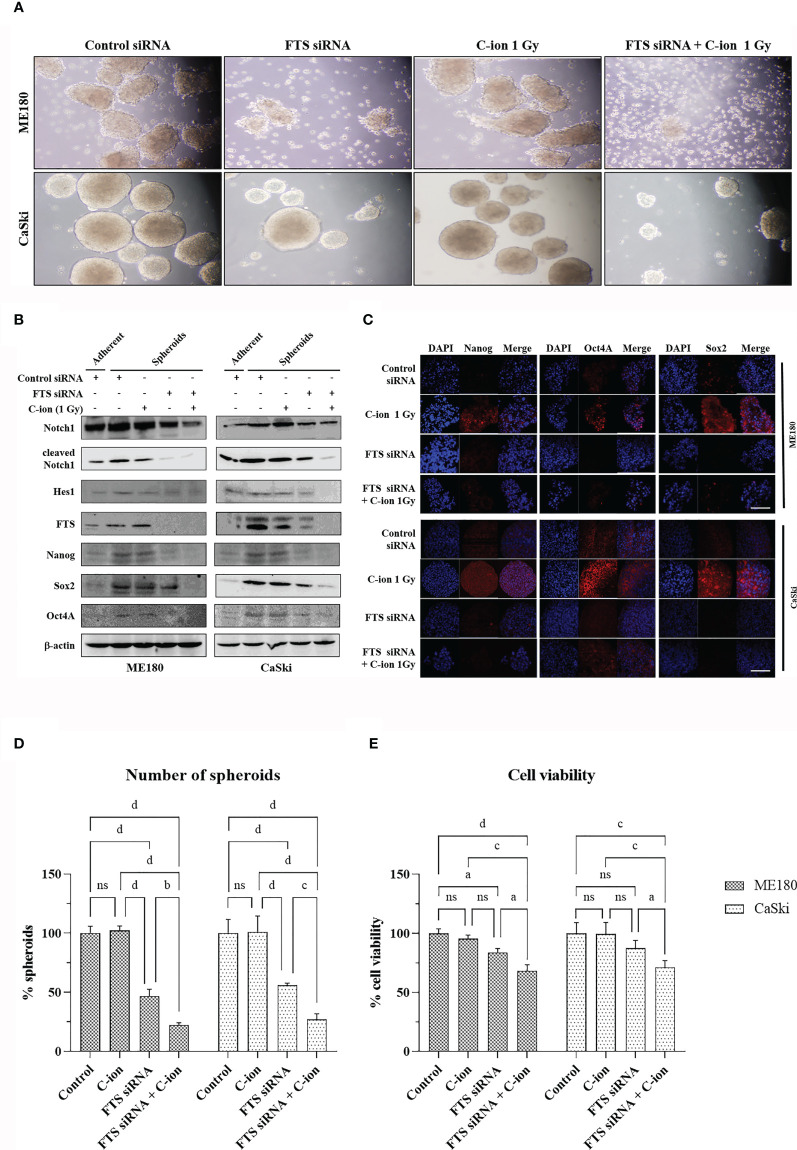
FTS silencing combined with C-ion reduces spheroid formation and cancer stem cell markers in cervical cancer cells. FTS intact or silenced ME180 and CaSki cells were irradiated with 0 or 1 Gy of C-ion. Following irradiation, the cells were harvested and cultured in ultra-low attachment plates for seven days. Spheroid formation assay **(A)**. Western blots and immunofluorescence performed with the spheroids displaying the reduced expression of stem cell markers in the FTS silenced group **(B, C)**, white bar in each panel corresponds to 50 µm. **(D)** shows the mean number of spheroids from five randomly selected fields under the microscope in each treatment group. Bars represent normalized values against control group, error bars represent ± SD. **(E)** shows the viability of the spheroid forming cells in each treatment group. Bars represent normalized values against control group, error bars represent ± SD. P values of < 0.05 were considered statistically significant. a p < 0.05; b p < 0.005; c p < 0.001; d p < 0.0001; ns, not significant.

**Figure 6:**
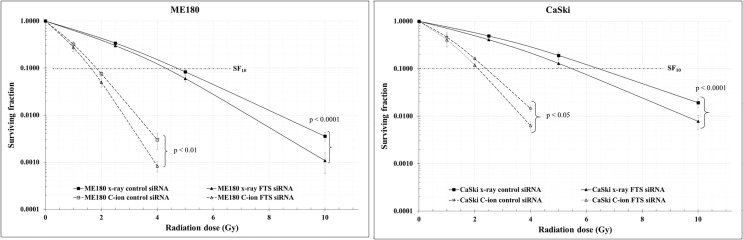
Clonogenic cell survival was reduced by the combination of radiation with FTS silencing. Cells treated with scrambled siRNA or FTS siRNA were irradiated with C-ion (0, 1, 2 and 4 Gy) or x-rays (0, 2.5, 5 and 10 Gy). Following irradiation, the cells were trypsinized, harvested and seeded in triplicates, in appropriate cell densities in 60 mm dishes. The cells were allowed to grow for 7-8 days. Colonies with 50 or more cells were considered as survivors. The survival curves represent the mean of three independent experiments ± SD.

The spheroid formation and the expression of stem cell marker proteins (OCT4A, SOX2 and Nanog) in the two cell lines were not changed by C-ion alone. However, they were decreased dramatically when combined with FTS-silencing (**Figures 5A–C** and **Supplementary Figure S5**). The number of spheroids and the viable cells in the spheroids were also reduced significantly by the combination of FTS-silencing and C-ion (**Figures 5D, E**). The RBE value of C-ion to x-rays has been generally considered to be about 2.5, hence we chose 1, 2 and 4 Gy of C-ion to compare the radiation effects with 2.5, 5 and 10 Gy doses of x-ray. The survival curves by C-ion or x-rays were significantly lowered when combined with FTS silencing (**Figure 6**). Silencing of FTS was seen to reduce the radiation dose by approximately 9.3% (x-rays) and 11.8% (C-ion) in ME180 cells, whereas 17.2% (x-rays) and 9.5% (C-ion) in CaSki cells (**Supplementary Table T1**). Therefore, the DMF at SF_10_ for ME180 cells was 1.093 (x-ray) and 1.118 (C-ion), whereas it was 1.172 (x-rays) and 1.095 (C-ion) for CaSki cells. We also calculated the RBE at SF10 for both the cell lines which were 2.58 for ME180 and 2.66 for CaSki in FTS intact group, while in the FTS silenced group it was 2.64 for ME180 and 2.49 for CaSki. We observed changes in the RBE value of C-ion by approximately 15.52% in ME180 cells and 16.72% in CaSki cells under the influence of FTS silencing (**Supplementary Table T1**).

## Discussion

In the current study, the expression of FTS and Notch signaling molecules (Notch 1 2, 3, cleaved Notch1 and Hes1) was upregulated, dose dependently, in response to C-ion in CaSki cells (**Figure 1** and **Supplementary Figure S2**). Additionally, upregulation of γ-secretase complex molecules (presenilin1, presenilin2, nicastrin and PEN2) was also observed in a radiation dose and time dependent manner (**Figure 2** and **Supplementary Figure S3**). The notch signaling pathway is well-known for its vital role in regulating cell division, differentiation, survival and maintenance of the CSC population in many human cancers, including cervical cancer (25, 29–32). RT awakens CSCs to lead tumor relapse and subsequent metastasis, although little is known about the underlying mechanism (33). The radioresistance of CSCs is governed by a few extrinsic factors (hypoxia, tumor microenvironment, etc.) and intrinsic factors (reactive oxygen species, DNA repair, apoptosis, autophagy, cell cycle status, etc.). Activation of Notch signaling leads to treatment failure after RT. Theys et al. reported that radiation induces upregulation of Notch signaling in non-small cell lung cancer (NSCLC) *in vitro* (34). Higher Notch signaling has been shown to accelerate tumor growth and increased radioresistance in NSCLC *in vivo* (35). Therefore, targeting the Notch signaling pathway could be an effective therapeutic approach to overcome radioresistance (11). The γ-secretase complex is a multi-subunit enzyme that plays an important role in the cleavage of intramembrane substrates of Notch receptors. The cleaved Notch1 is accountable for inducing the genomic functions of Notch signaling. Inhibition of γ-secretase has been reported to enhance radiosensitivity *via* blocking the Notch signaling pathway (36). γ-Secretase inhibitors (GSIs) in combination with radiation may prevent up-regulation of the Notch receptor, ligand and other family members and consequently diminish the number of surviving CSCs (37). Therefore γ-secretase inhibitors are now being studied in many clinical trials against colorectal cancer, breast cancer, melanoma, glioma and lung cancer (37). This study observed that silencing of FTS alone or in combination with C-ion attenuated overexpression of Notch and γ-secretase complex molecules (**Figure 3** and **Supplementary Figure S4**).

In adherent cervical cancer cells FTS co-precipitated with Notch1/cleaved Notch1/Hes1, suggesting FTS molecular interaction with Notch molecules (**Figure 4A**). IF assay demonstrated FTS co-localization with Notch1/cleaved Notch1/Hes1 in both the cell lines. FTS silencing not only diminished their expression but also prominently reduced the co-localization (**Figures 4B–D**). The co-localization of Notch1/cleaved Notch/Hes1 and FTS spectacles the importance of FTS in mediating the Notch signaling in cervical cancer cells. We have previously identified putative residues involved in the interaction between FTS and Notch by *in silico* molecular docking (10), which further strengthens our IP and IF findings. CSCs are in a quiescent state in most of the established tumors. Their innate radioresistance helps them survive the radiation exposure more easily as compared to differentiated cancer cells. Recent evidences show that CSCs play a crucial role in recurrence and metastasis in many cancers after radiotherapy (33). It has been reported that following radiation, CSCs are enriched both *in vitro* and *in vivo*, indicating towards the possibility of radiation-induced generation of CSCs (38). Formation of spheroids is a hallmark of cancer stem cells, therefore in the present study, we evaluated spheroid formation in the cervical cancer cells and compared expression levels of Notch and its target protein Hes1, in addition to cancer stem cell markers. In this study, we report that 1 Gy C-ion does not affect the spheroid formation ability of ME180 and CaSki cells. Our finding is consistent with other reports, where sphere-type cells were found to be resistant to both x-rays and C-ion beams (39). Interestingly, FTS silencing alone or in combination with 1 Gy C-ion inhibited spheroid formation in both the cell lines; however, spheroid inhibition was more remarkable in ME180 cells (**Figures 5A, D**). Similarly, the cell viability of spheroids was unchanged with 1 Gy C-ion, but it was reduced by FTS silencing alone or in combination with C-ion (**Figure 5E**). Upregulation of SOX2 and OCT4A indicates radiation resistance in cervical cancer cells (40). The stem cell signaling molecules (Notch related) were overexpressed in cervical cancer adherent cells as a result of 1 Gy C-ion (**Figure 1**), but there was no change in the expression level of these molecules along with other cancer stem cell markers (Nanog, SOX2 and OCT4A) in the spheroid populations (**Figures 5B, C** and **Supplementary Figure S5**). Nonetheless, a significant attenuation of Notch molecules and cancer stem cell markers was observed upon FTS silencing, indicating a potential role of FTS in the cancer stem cell signaling pathway (**Figures 5B, C** and **Supplementary Figure S5**). We finally compared DMF at SF_10_ after irradiation with C-ion and x-ray in FTS intact and silenced cells. Both the cell lines exhibit significantly increased radiation sensitivity upon FTS silencing (ME180-x-ray: p value <0.0001, C-ion: p value <0.01; CaSki – x-ray: p value <0.0001; C-ion: p value <0.05). At SF_10_ doses of C-ion and x-ray (**Figure 6** and **Supplementary Figure S6, Supplementary Table T1**) a reduction of approximately 10% radiation dose was observed in the FTS silenced group. These findings suggest radiosensitization can be achieved both in C-ion and x-ray by FTS-silencing.

Although few reports on C-ion therapy show eradication of CSC in glioma/cancer (41), data pertaining to the Notch signaling pathway by C-ion therapy are not available. This is the first report to show that FTS silencing combined with C-ion targets the Notch signaling and reduces the spheroid formation, cancer stem cell markers and clonogenic survival in cervical cancer cells.

## Author’s Note

PDS, PKC and W-YP performed the experiments described in this manuscript at NIRS, Japan, as visiting researchers.

## Data Availability Statement

The raw data supporting the conclusions of this article will be made available by the authors, without undue reservation.

## Author Contributions

PDS: conceptualization, methodology, investigation, writing - original draft. PKC: methodology, investigation, writing - review and editing. TS: project administration, resources, supervision. K-HK: writing - review and editing. W-YP: visualization, supervision, writing - review and editing, project administration, resources and funding acquisition. All authors contributed to the article and approved the submitted version.

## Funding

This work was supported by the National Research Foundation of Korea (NRF-2016K1A3A7A09005582, 2018M7A1A1072274, 202011B36-04).

## Conflict of Interest

The authors declare that the research was conducted in the absence of any commercial or financial relationships that could be construed as a potential conflict of interest.

## Publisher’s Note

All claims expressed in this article are solely those of the authors and do not necessarily represent those of their affiliated organizations, or those of the publisher, the editors and the reviewers. Any product that may be evaluated in this article, or claim that may be made by its manufacturer, is not guaranteed or endorsed by the publisher.

## Abbreviations

CCRT, concurrent chemo-radiotherapy; C-ion, Carbon ion; CSCs, cancer stem cells; DAPI, 4′,6-diamidino-2-phenylindole; DMF, dose modifying factor; ECL, enhanced chemiluminescence; EGF, epidermal growth factor; FTS, fused toes homolog; GSI, γ-secretase inhibitors; Gy, Gray; h, hour; HES1, hairy and enhancer of split-1; HEY, HES related with YRPW motif; HIMAC, Heavy Ion Medical Accelerator in Chiba; HNSCC, head and neck squamous cell carcinoma; HPV, Human papillomavirus; IF, immunofluorescence; IP, immunoprecipitation; LET, linear energy transfer; NICD, Notch intracellular domain; OCT4A, Octamer-binding transcription factor 4A; PBS, phosphate-buffered saline; *PEN-2*, presenilin enhancer 2; RT, radiation therapy; SF, surviving fraction; SOX2, SRY-Box Transcription Factor 2.
