# Supplementary material for: Silencing of Fused Toes Homolog (FTS) Increases Radiosensitivity to Carbon-Ion Through Downregulation of Notch Signaling in Cervical Cancer Cells
Source: Front Oncol. 2021 Oct 26;11:730607. doi: 10.3389/fonc.2021.730607 (PMC8576531; doi:10.3389/fonc.2021.730607)
Supplement: Supplementary file 1 [file Presentation_1.pptx]

## Slide 1
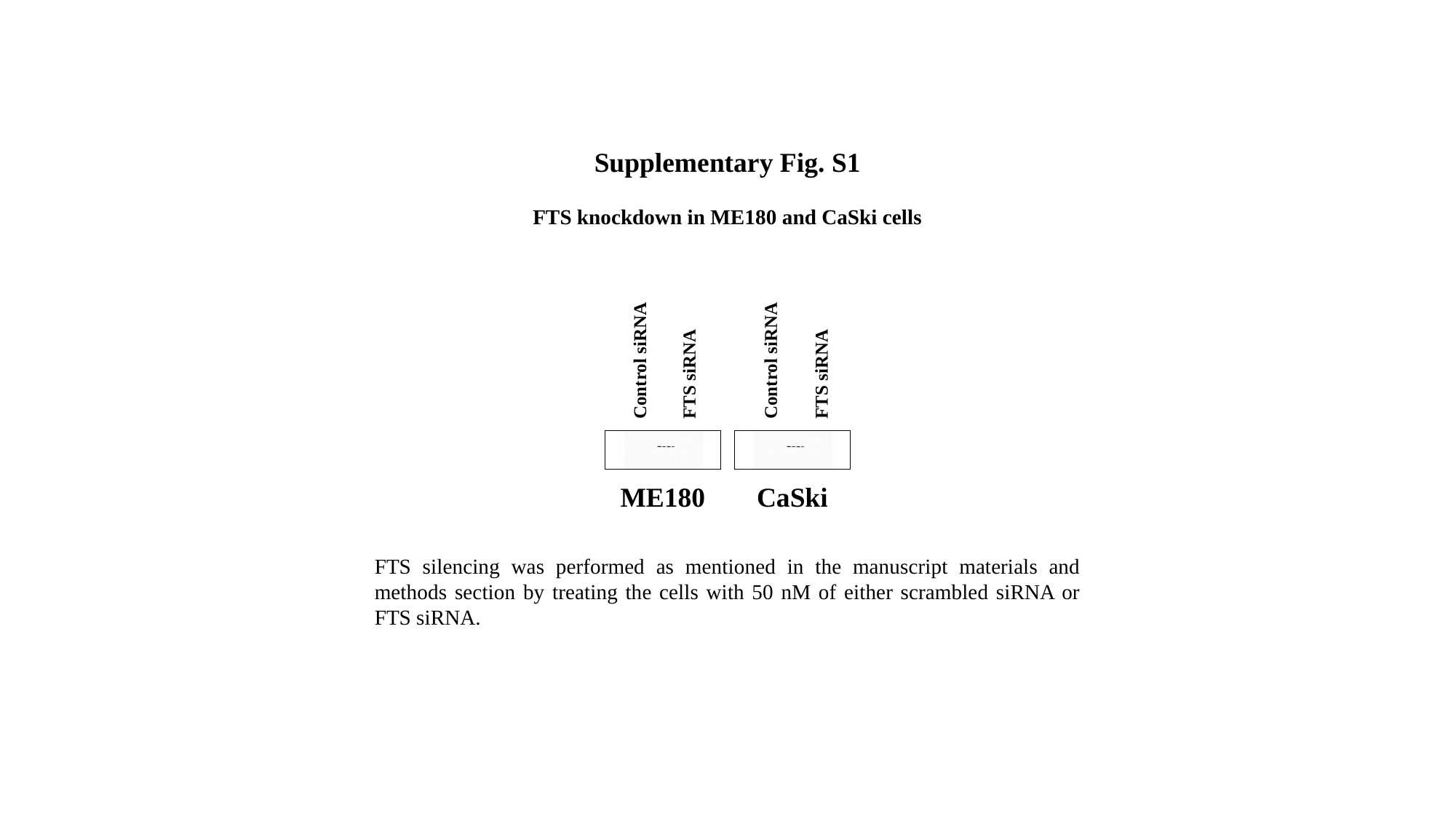

Supplementary Fig. S1
FTS knockdown in ME180 and CaSki cells
Control siRNA
FTS siRNA
ME180
Control siRNA
FTS siRNA
CaSki
FTS silencing was performed as mentioned in the manuscript materials and methods section by treating the cells with 50 nM of either scrambled siRNA or FTS siRNA.

## Slide 2
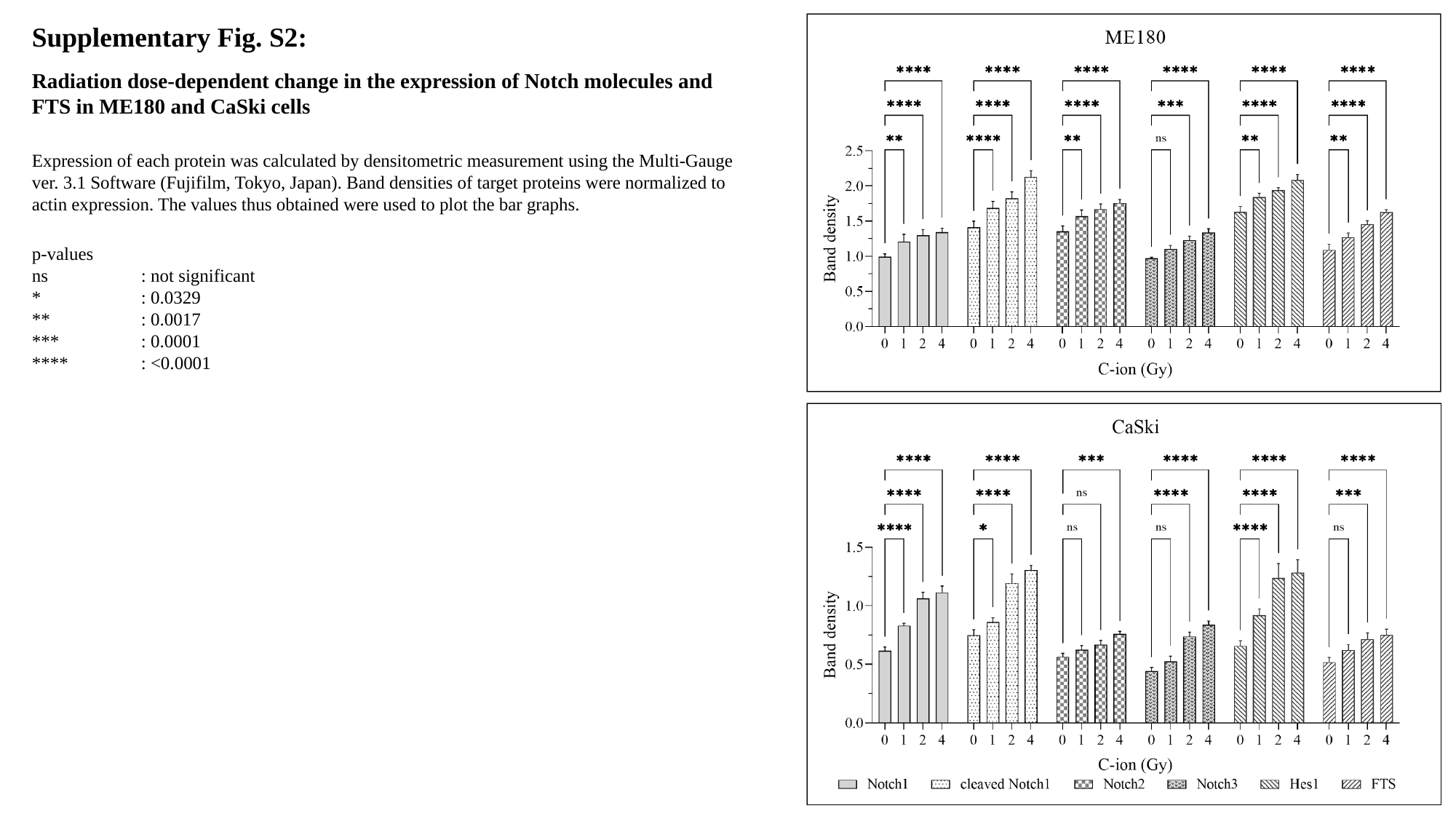

Supplementary Fig. S2:
Radiation dose-dependent change in the expression of Notch molecules and FTS in ME180 and CaSki cells
Expression of each protein was calculated by densitometric measurement using the Multi-Gauge ver. 3.1 Software (Fujifilm, Tokyo, Japan). Band densities of target proteins were normalized to actin expression. The values thus obtained were used to plot the bar graphs.
p-values
ns	: not significant
*	: 0.0329
**	: 0.0017
***	: 0.0001
****	: <0.0001

## Slide 3
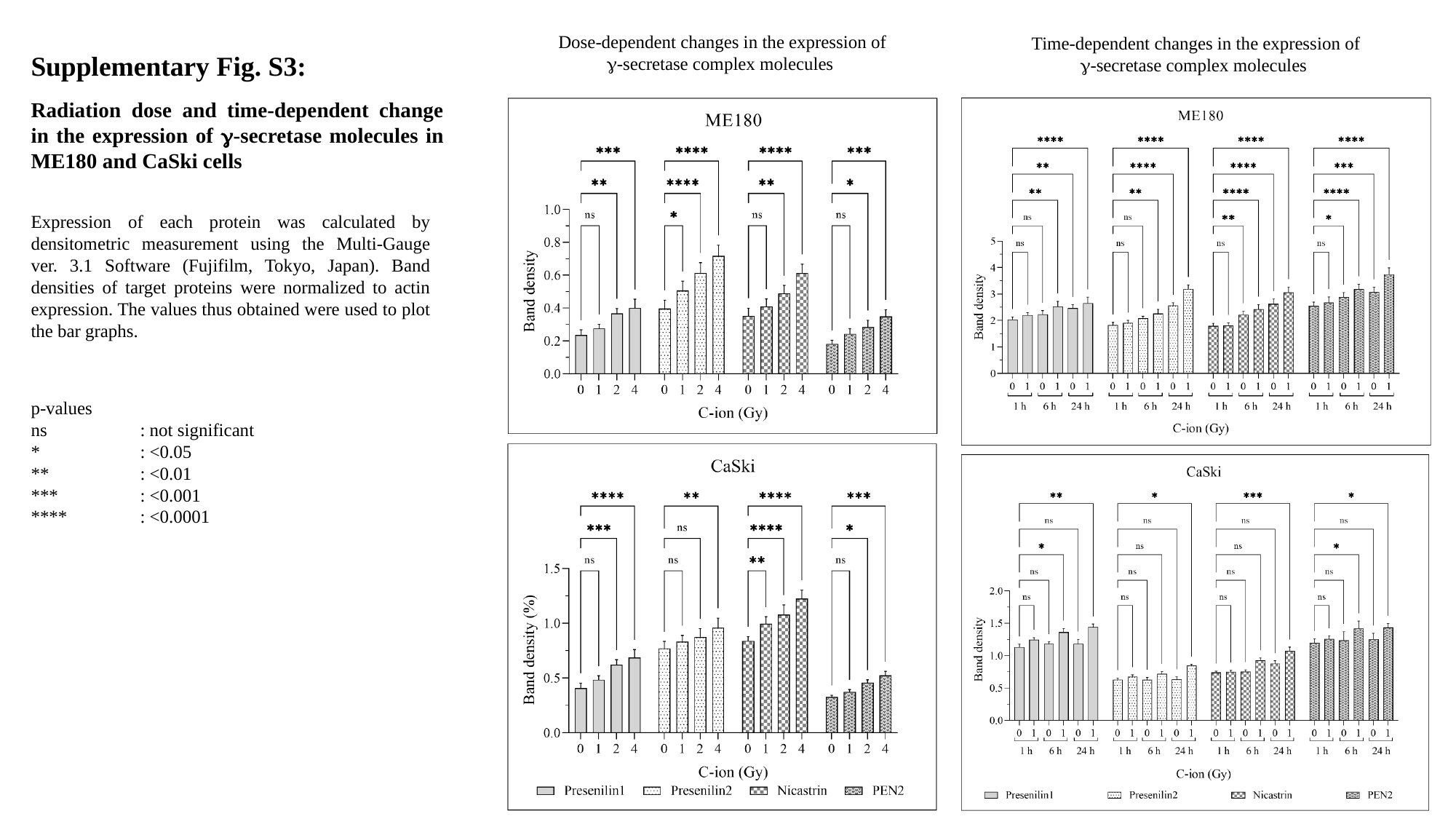

Dose-dependent changes in the expression of γ-secretase complex molecules
Supplementary Fig. S3:
Radiation dose and time-dependent change in the expression of γ-secretase molecules in ME180 and CaSki cells
Time-dependent changes in the expression of γ-secretase complex molecules
Expression of each protein was calculated by densitometric measurement using the Multi-Gauge ver. 3.1 Software (Fujifilm, Tokyo, Japan). Band densities of target proteins were normalized to actin expression. The values thus obtained were used to plot the bar graphs.
p-values
ns	: not significant
*	: <0.05
**	: <0.01
***	: <0.001
****	: <0.0001

## Slide 4
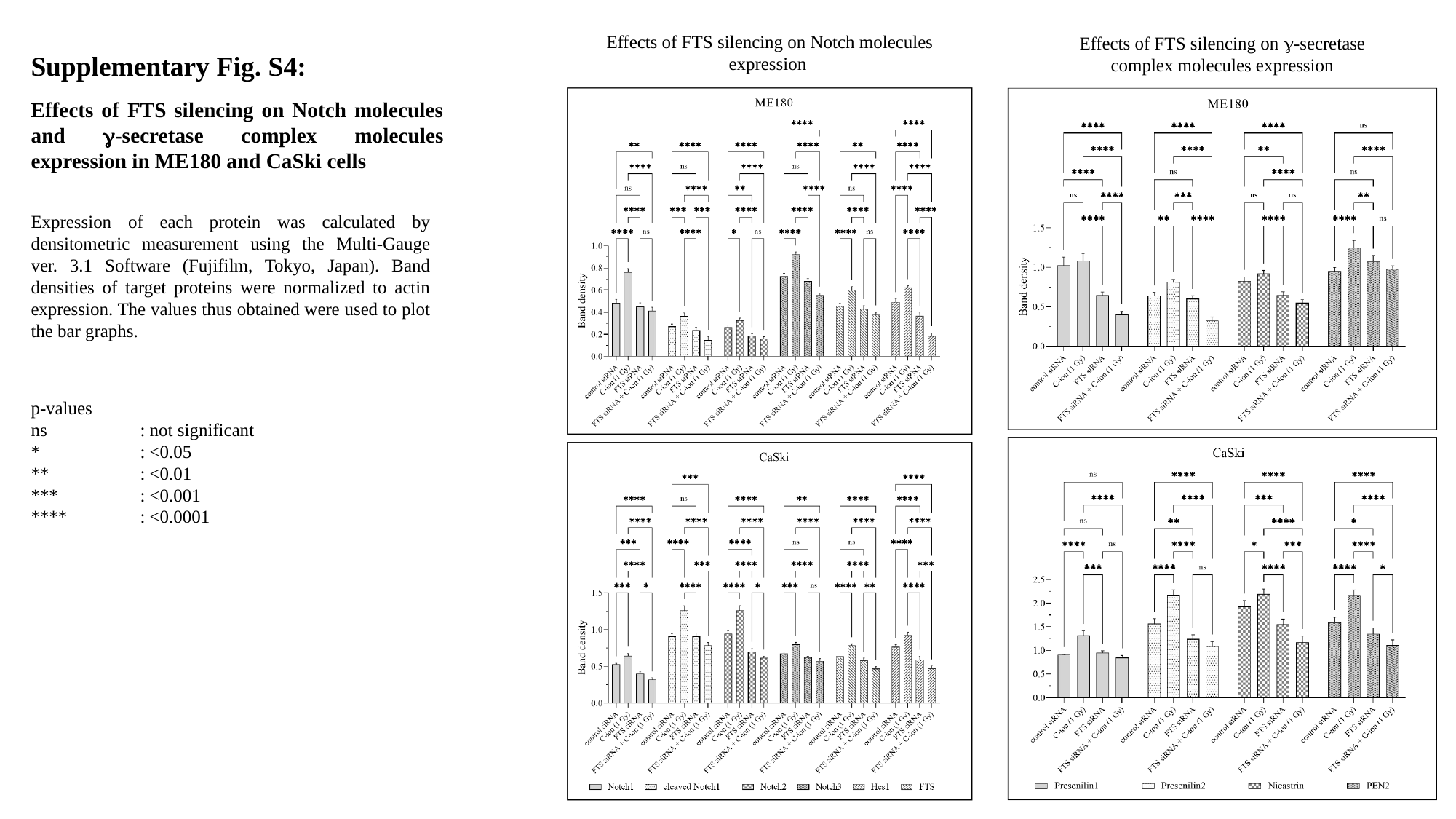

Effects of FTS silencing on Notch molecules expression
Supplementary Fig. S4:
Effects of FTS silencing on Notch molecules and γ-secretase complex molecules expression in ME180 and CaSki cells
Effects of FTS silencing on γ-secretase complex molecules expression
Expression of each protein was calculated by densitometric measurement using the Multi-Gauge ver. 3.1 Software (Fujifilm, Tokyo, Japan). Band densities of target proteins were normalized to actin expression. The values thus obtained were used to plot the bar graphs.
p-values
ns	: not significant
*	: <0.05
**	: <0.01
***	: <0.001
****	: <0.0001

## Slide 5
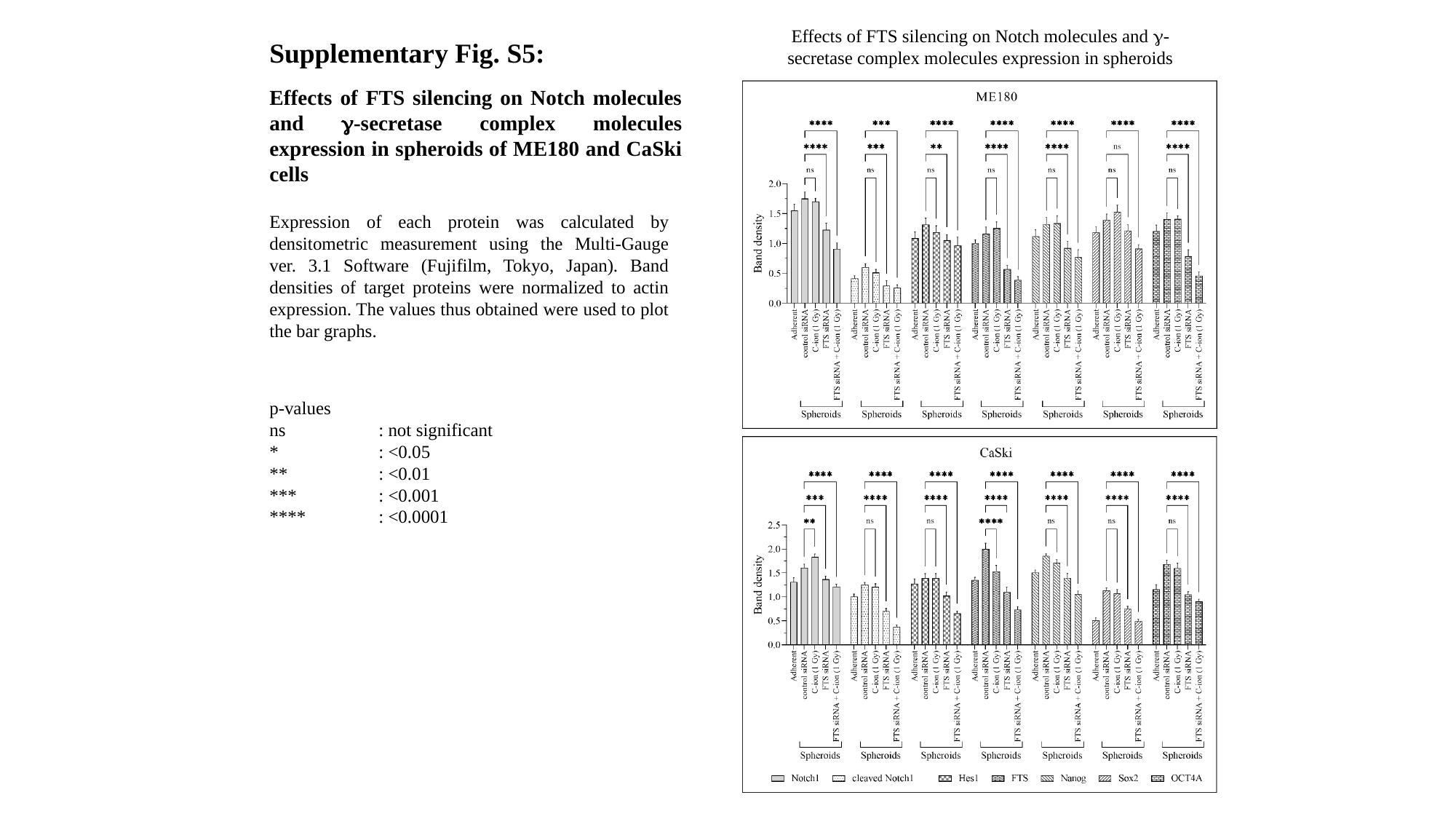

Effects of FTS silencing on Notch molecules and γ-secretase complex molecules expression in spheroids
Supplementary Fig. S5:
Effects of FTS silencing on Notch molecules and γ-secretase complex molecules expression in spheroids of ME180 and CaSki cells
Expression of each protein was calculated by densitometric measurement using the Multi-Gauge ver. 3.1 Software (Fujifilm, Tokyo, Japan). Band densities of target proteins were normalized to actin expression. The values thus obtained were used to plot the bar graphs.
p-values
ns	: not significant
*	: <0.05
**	: <0.01
***	: <0.001
****	: <0.0001

## Slide 6
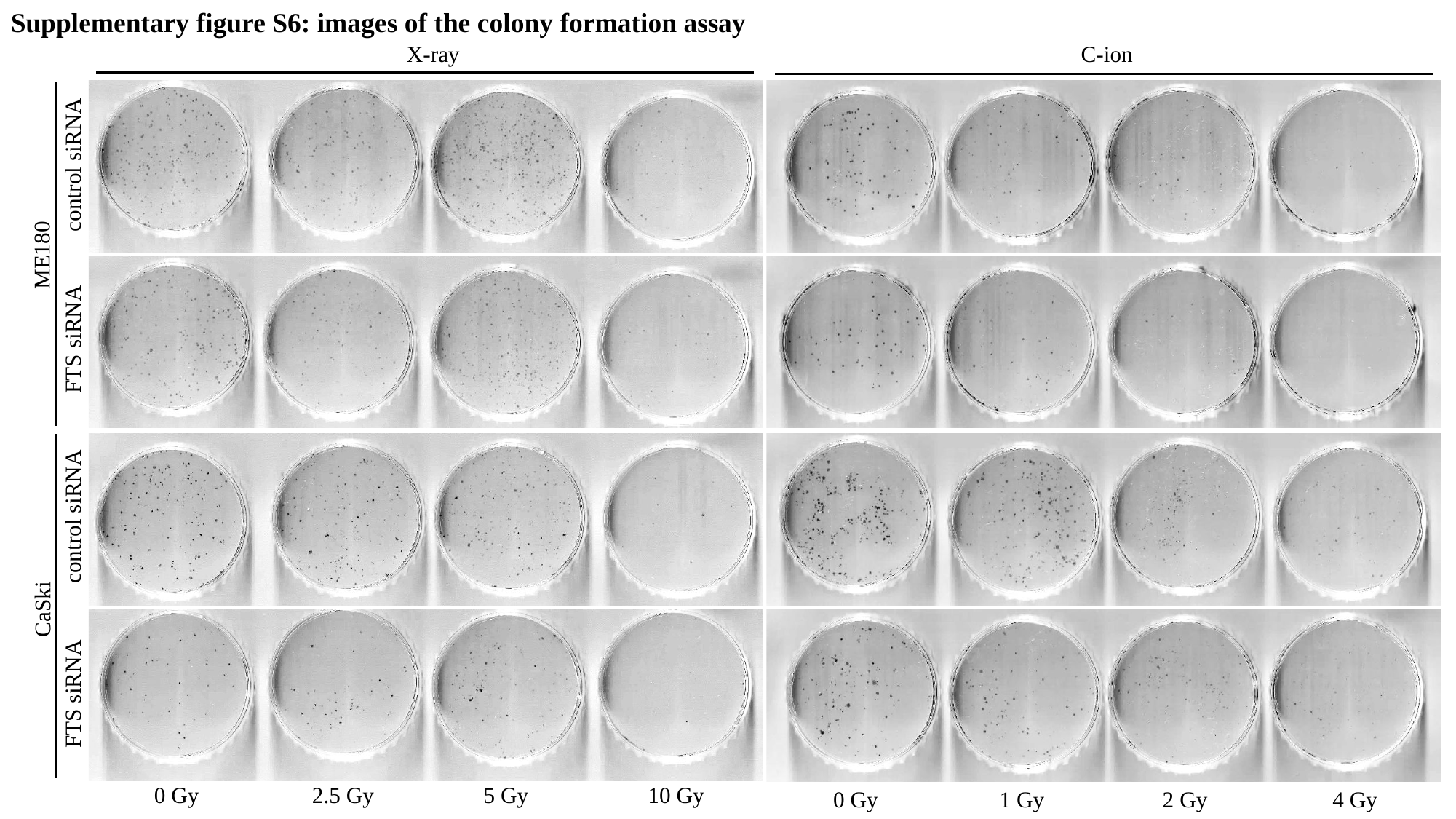

Supplementary figure S6: images of the colony formation assay
X-ray
control siRNA
FTS siRNA
control siRNA
FTS siRNA
0 Gy
2.5 Gy
5 Gy
10 Gy
ME180
CaSki
C-ion
0 Gy
1 Gy
2 Gy
4 Gy

## Slide 7
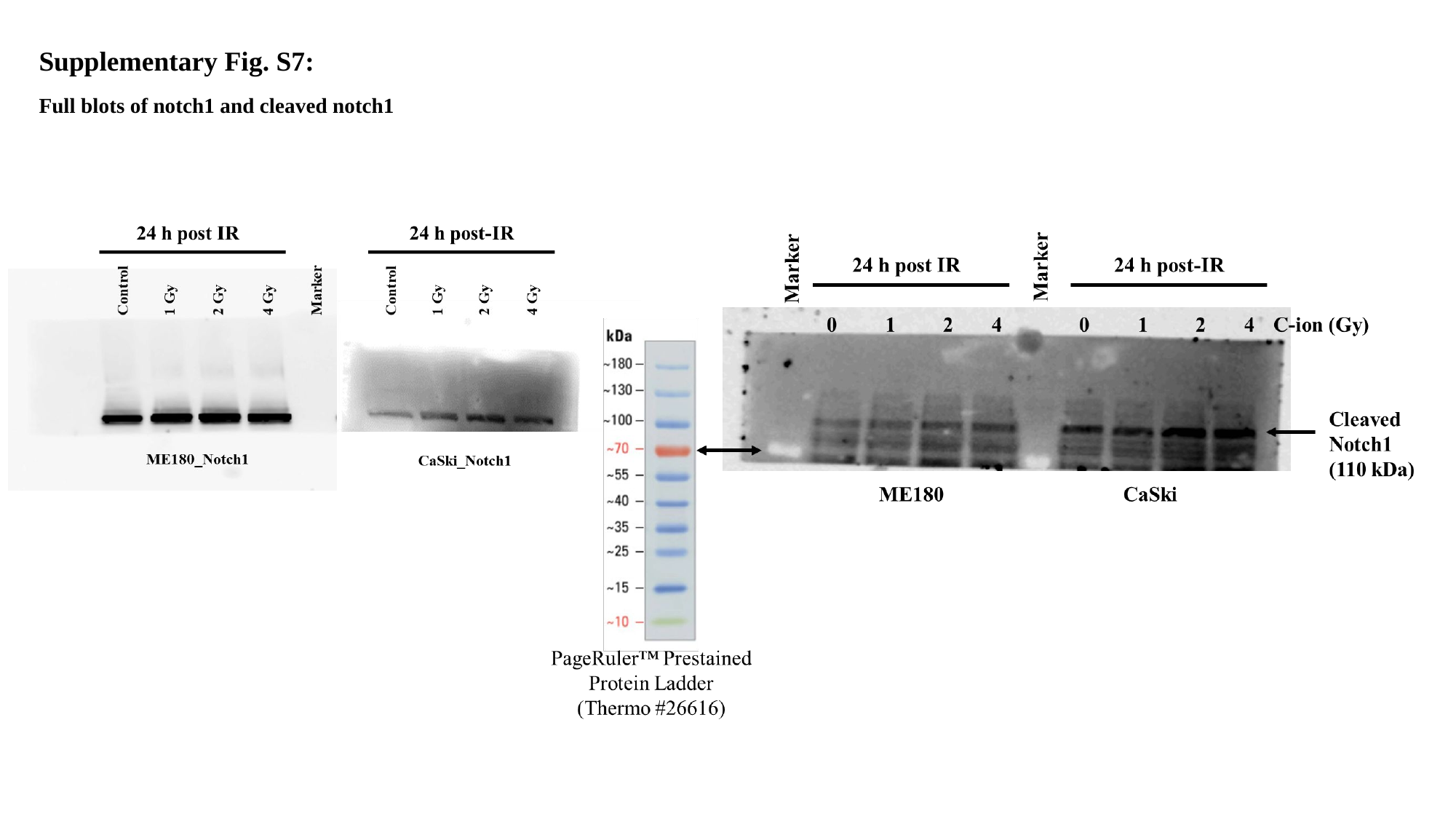

Supplementary Fig. S7:
Full blots of notch1 and cleaved notch1

## Slide 8
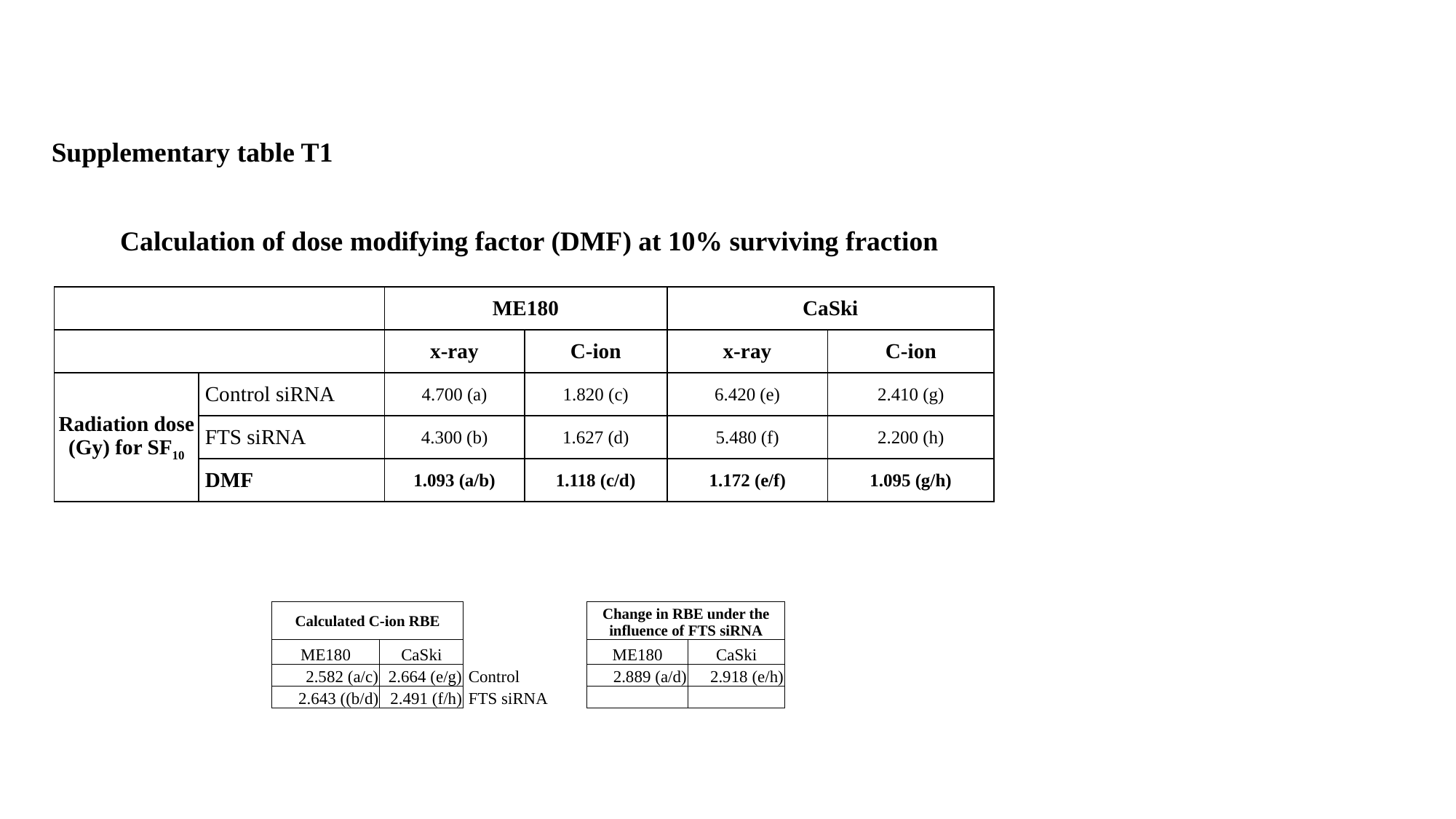

Supplementary table T1
Calculation of dose modifying factor (DMF) at 10% surviving fraction
| | | ME180 | | CaSki | |
| --- | --- | --- | --- | --- | --- |
| | | x-ray | C-ion | x-ray | C-ion |
| Radiation dose (Gy) for SF10 | Control siRNA | 4.700 (a) | 1.820 (c) | 6.420 (e) | 2.410 (g) |
| | FTS siRNA | 4.300 (b) | 1.627 (d) | 5.480 (f) | 2.200 (h) |
| | DMF | 1.093 (a/b) | 1.118 (c/d) | 1.172 (e/f) | 1.095 (g/h) |
| Calculated C-ion RBE | | | Change in RBE under the influence of FTS siRNA | |
| --- | --- | --- | --- | --- |
| ME180 | CaSki | | ME180 | CaSki |
| 2.582 (a/c) | 2.664 (e/g) | Control | 2.889 (a/d) | 2.918 (e/h) |
| 2.643 ((b/d) | 2.491 (f/h) | FTS siRNA | | |
